# Kinetic arrest during the drying of cellulose nanocrystal films from aqueous suspensions analogous to the freezing of thermal motions

**DOI:** 10.1038/s41598-022-24926-8

**Published:** 2022-12-05

**Authors:** Meng-Hsiang Chang, Masahito Oh-e

**Affiliations:** Institute of Photonics Technologies, Department of Electrical Engineering, National Tsing Hua University, 101 Sec. 2 Kuang-Fu Road, Hsinchu, 30013 Taiwan

**Keywords:** Liquid crystals, Liquid crystals, Molecular self-assembly, Molecular self-assembly, Soft materials, Fluids, Liquid crystals, Colloids

## Abstract

A comprehensive understanding of controlling the iridescence of cellulose films by manipulating the alignment and helical pitch of cellulose nanocrystals (CNCs) is required to advance cellulose photonics and its optoelectronic applications. Aqueous suspensions of CNCs exhibit a cholesteric liquid crystal (LC) phase with structural color; however, attaining a uniformly colored film is extremely difficult. Presumably, because multiple interrelated factors influence the CNC molecular alignment and helical pitch, existing models are not necessarily conclusive and remain a subject of debate. To eventually achieve homogeneously colored films, we compare aqueous CNC suspensions as a lyotropic liquid LC with thermotropic ones, and we spectroscopically confirm that the coloration of CNC droplets originates from the periodic CNC structure. The suspension drying process significantly influences the quality of iridescence of CNC films. Rapidly drying a droplet of a CNC suspension forms a concentric rainbow film, with red edges and a blue center, typical of the coffee-ring effect observed in air-dried films. By contrast, slow drying under controlled humidity, which reduces capillary flow, provides higher uniformity and a large blue area. Orbitally shaking films while drying under high humidity further improves the uniformity. Therefore, the evaporation rate significantly influences the thermodynamically stabilized helical pitch of CNCs, which determines the structural color. We qualitatively model the kinetic arrest induced by the rapid evaporation of lyotropic LCs in a manner equivalent to that induced by the rate of temperature change in thermotropic LCs and other materials.

## Introduction

Natural nanophotonic structures can be observed in nature, such as the coloration observed on living creatures^1–9^; however, fabricating artificial materials, such as polymers, that are equivalent to natural structures remains a challenge in terms of controlling the order of molecules on various scales from nanoscopic to macroscopic lengths^10–13^. Surprisingly, natural processes in biology can realize delicate molecular order and disorder across various length scales. Creating biomimetic polymers while utilizing their self-organizing nature is of significant interest because successfully controlling the routes of self-organization is crucial to realizing the scalable assembly of nano- and microstructured materials with the expected designed optical properties.

As high-value-added functional nanostructured materials, cellulose nanocrystals (CNCs) provide various unique material functions that have prompted us to employ them in a wide range of applications^13–17^. One of the most recent advances in optoelectronic applications using CNCs is cellulose pigments^18^, which have even been used to produce films of CNCs with large-scale ordering^19^. In further detail, self-assembled CNCs were first confined within emulsified microdroplets. Then, during the drying process, the microdroplets were likely subject to multiple buckling effects owing to the evaporation of the solvent and/or thermal post-treatments. The color of the pigment dispersions was believed to be caused by the degree to which the nanostructures contracted within the microdroplets during drying, and the coloration was experimentally confirmed to be structural color.

In general, CNCs are lightweight, rigid nanorod-shaped macromolecules with lengths of 100–200 nm and widths of 5–15 nm that can be biosourced from cotton or wood pulp and can form a stable colloidal suspension. In addition, above a threshold concentration, they can spontaneously self-assemble into a cholesteric liquid crystal (LC) phase^20,21^. This cholesteric structure commonly occurs in natural cellulose derived from plants^6^ and chitin tissues from crabs^22,23^ and insects^3^. The ability of CNCs to form cholesteric LCs has been investigated in terms of how to manipulate coloration in thin solid films^24–29^ and how to construct cellulosic or inorganic photonic sensors using nanotechnological methods^17,30–33^. Further, incorporating dopants such as fluorescent molecules or plasmonic gold nanorods into cholesteric structures gives rise to an additional controllable parameter, namely, whether their order is positional or orientational, in addition to an optical response induced by the chirality of CNC structures^34–37^.

However, these self-organizing structures usually lack long-range order; thus, polydomain structures frequently appear with defects, cracks, and misalignments between neighboring domains, resulting in a pixelated iridescent color rather than a uniform vibrant optical effect^38^. In laboratory settings, the molecular alignment order of self-organized CNC films that form from suspensions has been improved in different ways, e.g., using external fields. A strong magnetic field is known to enable the alignment of the CNC cholesteric domains owing to the inherent diamagnetic anisotropy of the nanorods^39,40^, thereby forming a single monodomain structure with the helicoid axes oriented along the magnetic field^41^. Although magnetic fields enable aligning the entire cholesteric structure with a slow relaxation time of several hours with respect to the kinetics of the alignment^42^, they do not allow a high degree of control over the periodicity.

Electric fields also make it possible for cellulose fibers in a nonpolar solvent to align parallel to the applied fields^43,44^. Using a CNC suspension diluted with a nonpolar solvent, Frka-Petesic et al. revealed that this alignment is caused by electric-field coupling with permanent and induced dipole moments on individual CNC molecules^45^. In addition, the electric and magnetic field-induced arrangement of other colloidal LCs has been extensively investigated^46–51^. By continuously applying magnetic fields, Dogic and Fraden^52^ caused colloidal LCs made of chiral fd viruses to convert from cholesteric to nematic. Meanwhile, an ionic concentration gradient was introduced using an applied DC electric field, which generated an electro-optical response in an aqueous solution of hydroxypropyl cellulose^53,54^, a polymeric cellulose derivative that forms lyotropic cholesteric phases. Similarly, controlling the iridescence of concentrated CNCs in a nonpolar solvent has been revealed to be a good way to demonstrate the efficacy of electric fields in aligning CNCs^55^.

These external fields indeed help align CNCs to some extent, but they do not necessarily ensure that CNC films remain uniform when they dry. Presumably, this is because some factors impede the uniformity of the films, for example, the strong self-assembling nature of CNCs in highly fluid suspensions. In addition, the evaporation process of the solvent, which is not a factor in thermotropic LCs, may deteriorate this uniformity. As a result, most dried CNC films contain a characteristic polydomain mosaic pattern instead of a uniform optical effect. Therefore, understanding these factors is important for attaining large-area, uniform dried CNC films.

Although hydrodynamic shear helps transform the cholesteric order into a nematic one^56,57^, Lagerwall et al.^58,59^ demonstrated that applying gentle circular shear while drying a suspension locally enhanced the vertical alignment of the cholesteric structures; however, the helicoidal order was gradually distorted toward the edges. Nonetheless, these simple non-static drying methods would be interesting to explore further, as they would provide some insights into attaining a uniform pattern over a large area. Lagerwall et al. also suggested that the initial CNC concentration of a suspension is key and thus treated it as a crucial factor, showing that a high CNC concentration that ensures a complete LC phase is preferable for obtaining uniform dried films over a large area^58^. Electron microscopy imaging of the fractured films confirmed the largely uniform orientation of the helical axes perpendicular to the film plane; however, when we attempted to employ a high CNC concentration that exhibited a complete LC phase, our colored film was hardly uniform. Presumably, high viscosity is another negative factor in this case. In summary, no universal methodology for obtaining uniform dried CNC films has been developed, and the best manner to achieve such films is still under debate.

Exploiting the lyotropic cholesteric LC phase using CNC suspensions is increasingly focused in numerous directions; however, realizing uniform CNC films is more complex than often perceived, leading to reproducibility issues and confusion. Our group has also encountered difficulties in producing uniformly colored CNC films from aqueous suspensions. Compared with those in thermotropic LCs, it is significantly more difficult to attain uniform molecular alignment and orientation in CNC suspensions; thus, we turn our attention to differences in the properties and processes between lyotropic and thermotropic LCs when making films. This back-to-basics approach reveals a distinctive feature of lyotropic LCs, i.e., the use of a solvent, which is water in the case of aqueous CNC suspensions. When drying these suspensions to fabricate films, water flows through the medium as it evaporates. This is the most likely factor impeding the uniformity of the dried CNC films.

Gray^60^ explored the iridescent nature of droplets of dried CNC suspensions while correlating this property with the “coffee-ring” stain typically observed in air-dried films^61^. By observing the two-dimensionally U-shaped height profiles of the dried CNC droplets across the diameter of the droplets, they concluded that the evaporation of solvent and its corollary mass transfer caused the concentration gradient across the ring, which was thought to be related to a color gradient, with the amount of longer wavelengths decreasing toward the center of the droplet^60^. The formation of iridescent films is hypothesized to occur through two key processes; one is an equilibrated change in pitch as the concentration of the chiral rod-like species increases, and the other is a kinetically controlled stage wherein a gel or glassy state sets in as the film dries^24^. Further research, however, will be useful to confirm this hypothesis, comprehensively understand these and other related phenomena, and gather detailed physical insights into the drying process that forms the iridescent nature of the CNC droplets.

In this study, we address how color uniformity can be attained in CNC films using a simple method, while finding consistent mechanisms underlying their formation, together with some relevant parameters that must be controlled. Although some techniques and mechanisms that provide uniform CNC films and iridescent coloration, respectively, have been reported sporadically, these findings are yet to be conclusive and remain a subject of debate. Presumably, this is because multiple interrelated factors influencing the molecular alignment and helical pitch of CNCs are involved. Therefore, we take a fairly primitive approach to simplify various observations while qualitatively modeling the process of making films from aqueous CNC suspensions by analogy to the processes occurring in thermotropic LCs and other materials. For our purposes, we deal with only the uniformity of films formed from simple CNC droplets; we thereby deduce some preferable conditions for uniform alignment and hence homogeneous color.

## Methods

### Materials and preparation

A suspension of CNCs with a high sulfur content (CNC-HS [(C_6_H_10_O_5_)_x_(C_6_H_9_O_4_SO_4_Na)_y_)]) (solid content: 20%) was purchased from Cellulose Lab; this CNC-HS suspension was obtained from the sulfuric acid hydrolysis of prehydrolyzed kraft dissolving pulp, which was first purified by removing most of the non-cellulosic components in the biomass, such as lignin, hemicellulose, fats and waxes, proteins, and inorganic contaminants. Specifically, hydrolysis was carried out using 62–64% sulfuric acid to remove the amorphous regions of the cellulose microfibrils, followed by centrifugation and dialysis to remove residual acid and salt. The reaction time and temperature were approximately 1–2 h and 44–65 °C, respectively. The CNC-HS nanorods had a length of 135 nm and width of 7 nm, and the aspect ratio was approximately 20. The sulfur content was measured using conductometric titration^62^ against NaOH and quantified as [S] ≈ 1.02 ± 0.08 wt%.

We used the CNC-HS suspension without further purification and diluted it from 20% to lower concentrations of 1–10 wt% using deionized (DI) water by mixing a definite quantity of the 20% CNC suspension and the desired amount of DI water using a high-speed homogenizer (T 10 basic ULTRA-TURRAX^®^, IKA) at 6,000 rpm for 60 min. Further, the mixed suspensions were sonicated for 15 min to remove bubbles from the highly concentrated suspensions.

To observe the phases of the suspensions as a function of the CNC concentration, rectangular borosilicate capillaries (Capillaries #3536-050, VitroCom) with inside dimensions of 0.3 (path length) × 10 (width) and a glass thickness of 0.3 mm were used. The tube was then filled with the sample and sealed with clay. The samples were settled to allow phases to separate in these capillary tubes for at least 48 h prior to all experiments.

**Figure 1:**
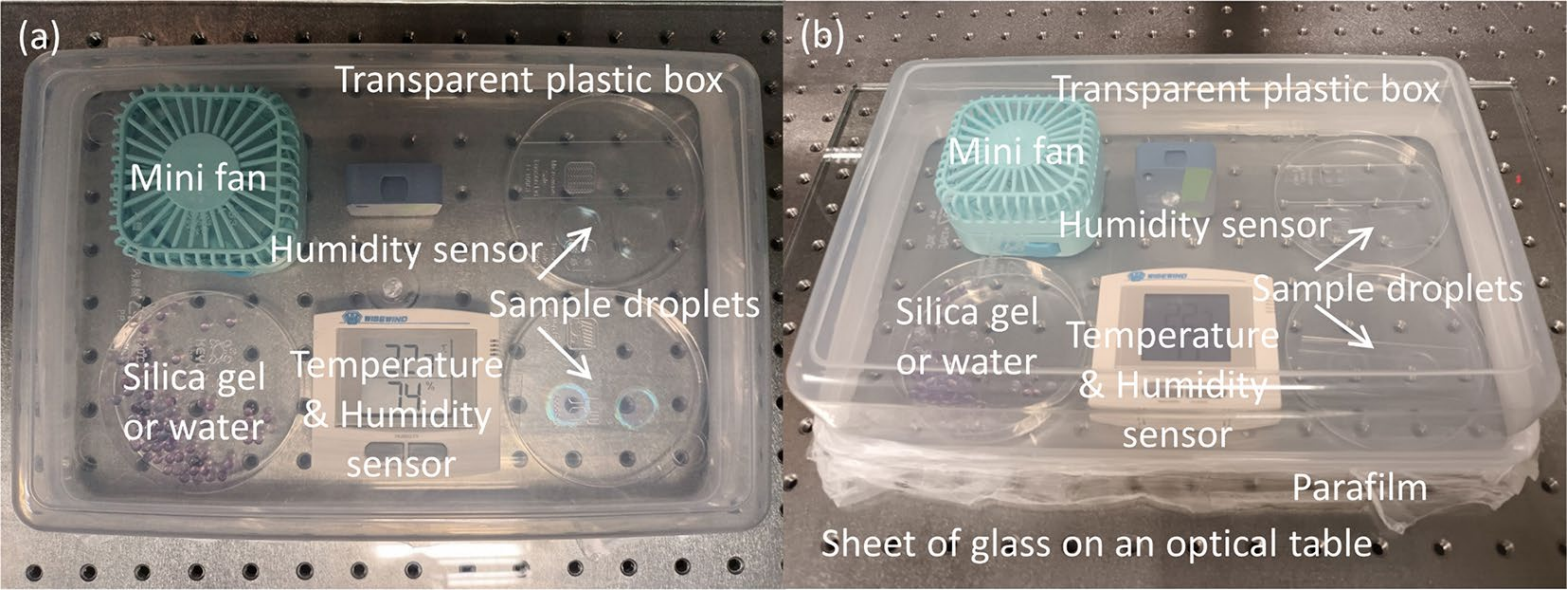
(**a**) Top and (**b**) side views of our typical setup for preparing CNC droplet films under humidity-controlled drying. Two humidity sensors were used to increase accuracy.

To conduct humidity-controlled drying, a humid atmosphere was created in a confined space using a transparent plastic box in a temperature-controlled laboratory at 24 °C. Figure 1 shows top and side views of the components in our typical setup for this drying method. A sheet of glass was placed on an optical table as a base to obtain a flat surface and to cap the holes of the optical table, and a transparent plastic box with dimensions of 20 × 10 × 5 cm covered the surface of the glass sheet. The confined space contained sample droplets with a total volume of 0.3 mL on glass slides, a humidity sensor (Model T1, tempi.fi) with a typical humidity accuracy of ± 3%, and a small fan. In addition, it contained a Petri dish with a diameter of 9 cm, in which either 0.1–10 mL of water or 10–40 g of silica gel (Tokai Chemical Industry Co., Ltd.) was placed around the samples in the confined space to control the humidity. Decreasing the humidity to < 20%, ~ 25%, and ~ 30% required ~ 40, ~ 20, and ~ 10 g of silica gel, respectively, which was placed in separate Petri dish. Meanwhile, the humidity was increased to ~ 75%, ~ 90%, and ~ 99% by adding 0.1, 1, and 10 mL of water in the Petri dish, respectively. The atmosphere in the confined space was homogenized by the small fan. In addition, the gap between the glass sheet and box was sealed with parafilm, which enabled creating a highly humid environment in the confined space.

For further analysis, some films were prepared under high-humidity conditions on an orbital shaker (S101, Firstek Scientific) to observe whether left-handed circular shear flow improved the color uniformity of the films^58^. The rotating speed could vary up to 150 rpm.

### Characterization

The phase separation of the suspensions was examined by observing the rectangular capillaries between crossed polarizers. The volume fraction of each phase in the total suspension was determined by measuring the height of each phase and that of the total suspension in the capillary tube. Polarized optical images were recorded using an Olympus BX53/BX53M-P polarizing optical microscope (POM), and a Nikon microscope SMZ745/SMZ745T was employed to investigate the optical properties of the films, primarily in reflection mode. The structural order of the sample was probed through its optical properties using several different microscope settings, including direct observation with and without a pair of crossed polarizers and light diffraction methods. The cholesteric pitch was estimated from the POM images of the fingerprint textures by counting 10 randomly chosen patterns in 10 or more POM images from different regions to obtain the average pitches and standard deviations. To characterize some points in the POM images, transmission and reflection spectra were recorded using an Ocean Optics HR4000 spectrometer.

## Results and discussion

Our first step was to observe the phase behavior of the CNC suspensions. When CNCs are suspended in water above a critical concentration, they can self-assemble into a lyotropic cholesteric LC phase^20,21^. The attractive and repulsive intermolecular interactions and the balance between them all play a role in governing the thermodynamic stability of a CNC colloidal suspension as well as its ability to self-assemble into an LC. Presumably, the attractive interactions are caused by van der Waals forces, whereas the repulsive forces originate from not only short-range steric repulsions but also longer-range steric or electrostatic ones^63^. Although at low concentrations, CNC nanorods orient rather randomly, thereby forming an isotropic phase, sufficiently high concentrations often locally promote intermolecular alignment between individual nanorods. Thus, when their concentration is increased, the samples must undergo a first-order phase transition while transitioning from an isotropic phase to a cholesteric phase, by way of an intermediate regime wherein both phases coexist. The ordered phase can be identified by characteristic cholesteric fingerprint patterns using a POM.

**Figure 2:**
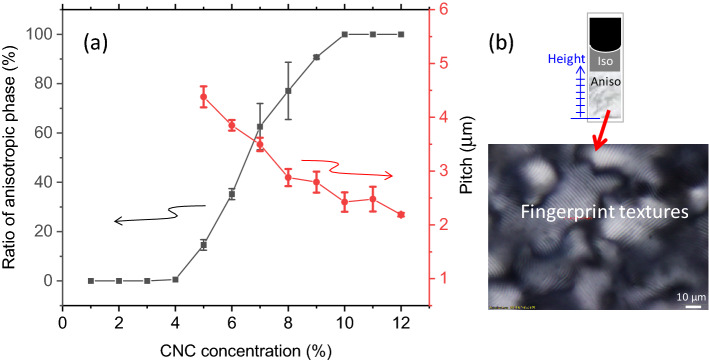
(**a**) Phase transition from isotropic to cholesteric (square dots) with the increasing CNC concentration and the corresponding equilibrium helical pitch (red circles). (**b**) Schematic of the height of the anisotropic phase with respect to that of the total suspension in the capillary tube (top) and the typical appearance of the anisotropic phase (bottom), exhibiting fingerprint textures, from which the pitch can be determined. The image was acquired using POM.

Figure 2a illustrates the phase behavior of CNC suspensions as a function of the CNC concentration, which exhibits a clear phase transition from pure isotropic to biphasic and anisotropic LCs with the increasing concentration, as expected. The phase diagram in Fig. 2b shows the proportion of the anisotropic phase as a function of the total CNC concentration in the suspension, which can be calculated as the ratio of the volume of the anisotropic phase to the total sample volume. In the biphasic regime, the suspension separates into two parts after the precipitate is allowed to settle in the tube: the upper phase is isotropic, and the lower one is anisotropic. This biphasic regime appears at CNC concentrations from ~ 4 to ~ 10%, beyond which the suspension exhibits only anisotropy.

In the LC phase, the cholesteric order of the CNCs is characterized by locally oriented nanorods along an average common direction^63^. The average orientation of molecules in a small volume is characterized as the director ${\varvec{n}}$, which spatially rotates about an axis ${\varvec{m}}$ and forms a helicoidal structure with respect to ${\varvec{m}}$. The CNCs assemble in the left-handed helical direction, which is caused by the chiral interactions between the nanorods; however, the intrinsic mechanism underlying the formation of the helicoidal structure is still under debate^64–67^. In a helicoidal structure formed by ${\varvec{n}}$, the distance required for ${\varvec{n}}$ to rotate a full 360° is defined as the helical pitch ${\varvec{P}}$. CNCs intrinsically generate optical properties when their helical pitches vary only within a certain range.

The helical structures of ${\varvec{n}}$ exhibit a characteristic “fingerprint texture” when the helical axis ${\varvec{m}}$ is parallel to the substrate surface, which means that the LCs must be vertically aligned to observe these fingerprint textures. Measuring the line distance of the fingerprint texture gives half of the helical pitch in the cholesteric LC phase. The texture observed through a POM is composed of planar and fingerprint textures, wherein the inclination of ${\varvec{m}}$ varies from orthogonal to parallel to the plane of the sample, with various disclinations. A POM can only be used to observe helical pitches from 2 to 5 µm; however, when the helical pitch approaches the wavelength of visible light, it becomes difficult to observe the fingerprint textures; thus, shorter pitches cannot be determined using this method.

Our purpose is to attain uniformly colored CNC films. To this end, the helical pitches values must be short enough to reflect visible light, and the alignment of the molecules must be controlled, which in turn controls helical axes; however, we have encountered difficulties in satisfying both requirements. The helical pitch of CNCs in aqueous suspensions is ~ 2 µm at most, as observed in Fig. 2, and the suspensions are colorless; therefore, even if we deposit a CNC film from these suspensions on a glass substrate via spin-coating or a dipping process, the film does not exhibit iridescence, and its color is rather transparent.

Thus, the helical pitch of CNC suspensions must be shortened to observe structural color in CNC films. In general, the pitch is affected by several factors involving different mechanisms, for example, adding a nonvolatile additive or cosolvent is known to influence the equilibrium pitch of cholesteric suspensions, and adding *D*-glucose leads to a blue shift^24^. However, the success of these previous strategies has not been reproducible in our laboratory, even with *D*-glucose and similar additives such as *D*-fructose and *D*-sucrose, and a reliable way to control the pitch has not yet been established and remains an area of active research^68^.

**Figure 3:**
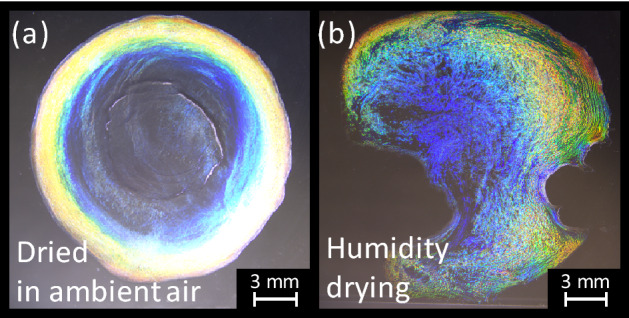
Comparison of two initial CNC droplets dried (**a**) in the ambient air and (**b**) under high humidity of approximately 70–80%. These droplets were made using the 7% CNC suspension. These images were acquired in reflection mode under white light without polarizers.

Fortunately, during the course of our experiments on phase transitions in CNC suspensions, we unexpectedly observed a partially colored film that had dried from a droplet of a CNC suspension that accidentally dripped onto a desk. Inspired by this coloration, we began to examine some intentionally made droplets from CNC suspensions. Figure 3 compares two cases of differently dried CNC suspension droplets: one droplet was dried into a film in the ambient air and the other under higher humidity (~ 70–80%), which tends to allow the droplets to deform during drying. In the former, multiple colors form a rainbow-like “coffee-ring” stain from red to blue from the outer to the inner region of the droplet, with a bluish color appearing to be more dominant and uniform in the film interior, the observation of which is consistent with ref 60. During the drying process of the CNC suspension droplets, these coffee-ring stains can form because of the capillary flow caused by contact line pinning^61,69^ (i.e., the tendency of the outline of the droplet to remain in place). Initially, the contact line of the droplet is pinned, and the CNCs are homogeneously distributed; however, as water evaporates at the pinned contact line, this evaporation induces capillary flow, which tends to draw the CNCs toward the perimeter while the contact line gradually recedes toward the center. This mechanism is how the coffee-ring effect is generally understood^60^. These colors can be observed without a pair of polarizers and therefore must be structural coloration derived from the various helical pitches of the CNCs, suggesting that their pitch differs between the suspension and the resulting films and that the pitch in suspension gradually shortens as the droplet evaporates.

**Figure 4:**
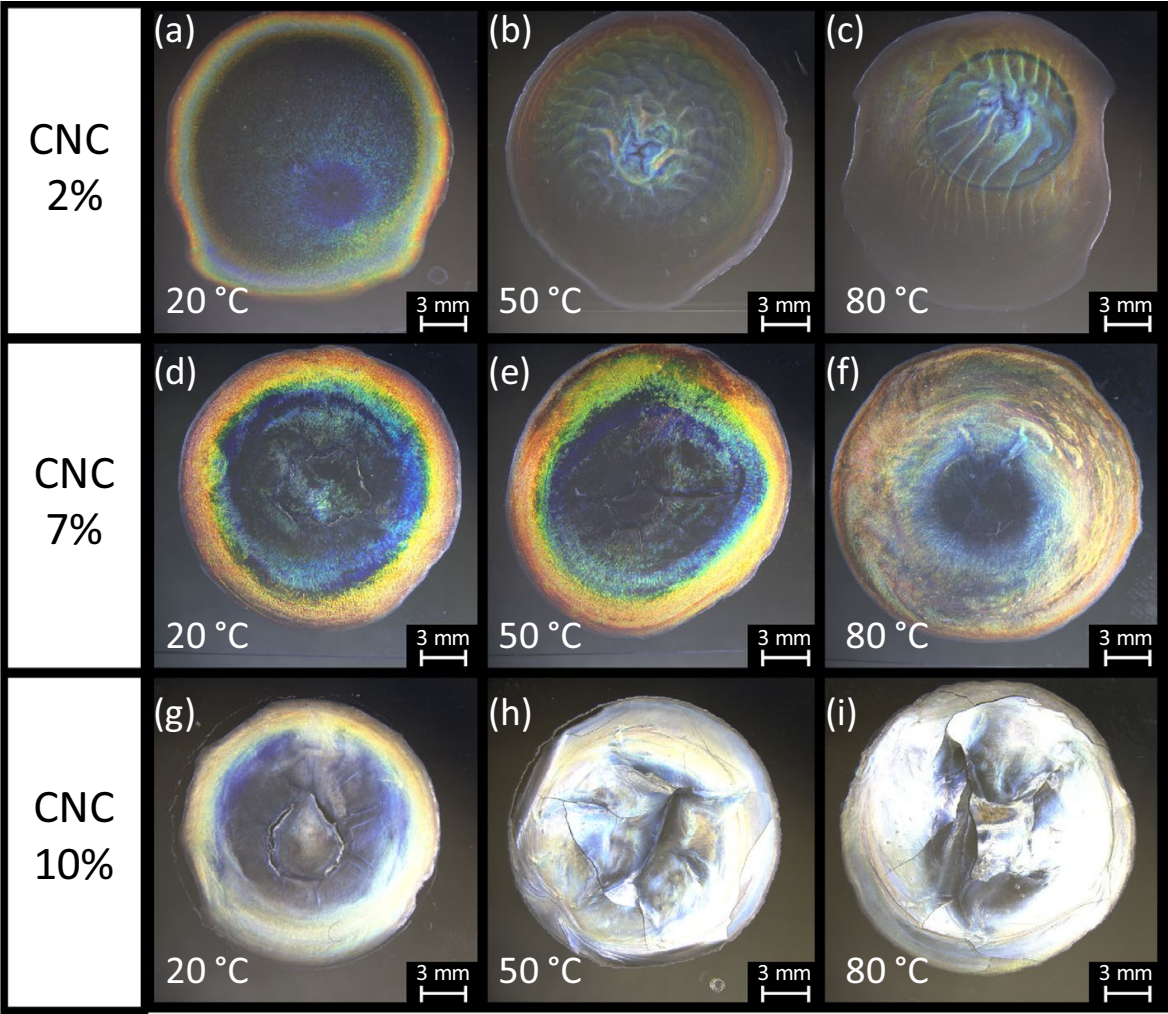
CNC droplets dried under various constant temperatures from suspensions of (**a**)‒(**c**) 2%, (**d**)‒(**f**) 7%, and (**g**)‒(**i**) 10% CNCs. These images were acquired in reflection mode under white light without polarizers.

Obviously, increasing the temperature promotes the evaporation of water from the droplets, which in turn makes the flow in the droplets more active. This more active flow during evaporation can be assumed to kinetically hinder the transition to a thermodynamically stable pitch, which would be equivalent to the kinetic arrest of molecular motions into a gel-like glassy state^68^. Further, even if the pitch falls into the visible range under this kinetic arrest, the resulting colors may eventually disperse owing to the disturbed flow in the droplets. Figure 4 compares the textures of some films made from droplets of aqueous CNC suspensions with different concentrations dried at various constant temperatures. The biphasic suspension, i.e., the 7% CNC suspension, provides the most iridescent films under our conditions. This result contradicts previous findings that a sufficiently high concentration to ensure full LC properties is preferable in order to promote a uniform helical orientation perpendicular to the film plane^58^. By contrast, with the 10% CNC suspension, remarkably bright whitish textures increasingly appear in the films as the temperature increases, and very weak coffee-ring stains appear after drying at room temperature (~ 20 °C). Further, some domains with dimensions ranging from submicrons to millimeters can be observed, presumably because another factor, such as viscosity and hence mass transfer, plays a role in forming a film as water evaporates from the suspension. The film of the 2% CNC suspension also exhibits coffee-ring stains after drying at room temperature, but they can reasonably be considered weaker than those in the film made from the 7% CNC suspension. Owing to the isotropic nature of the 2% CNC suspension, the major central part of the film is dark. At higher temperatures, lightly colored textures can be observed against the dark background, which resemble an activated trace of mass transfer. Overall, as the temperature increases, the colors in the films mix over a larger area, and mixed stains appear rather than coffee-ring stains, thus validating our predictions of active flow in the droplets based on existing knowledge. When dried at room temperature, however, the texture of the films changes from mixed stains to coffee-ring stains.

**Figure 5:**
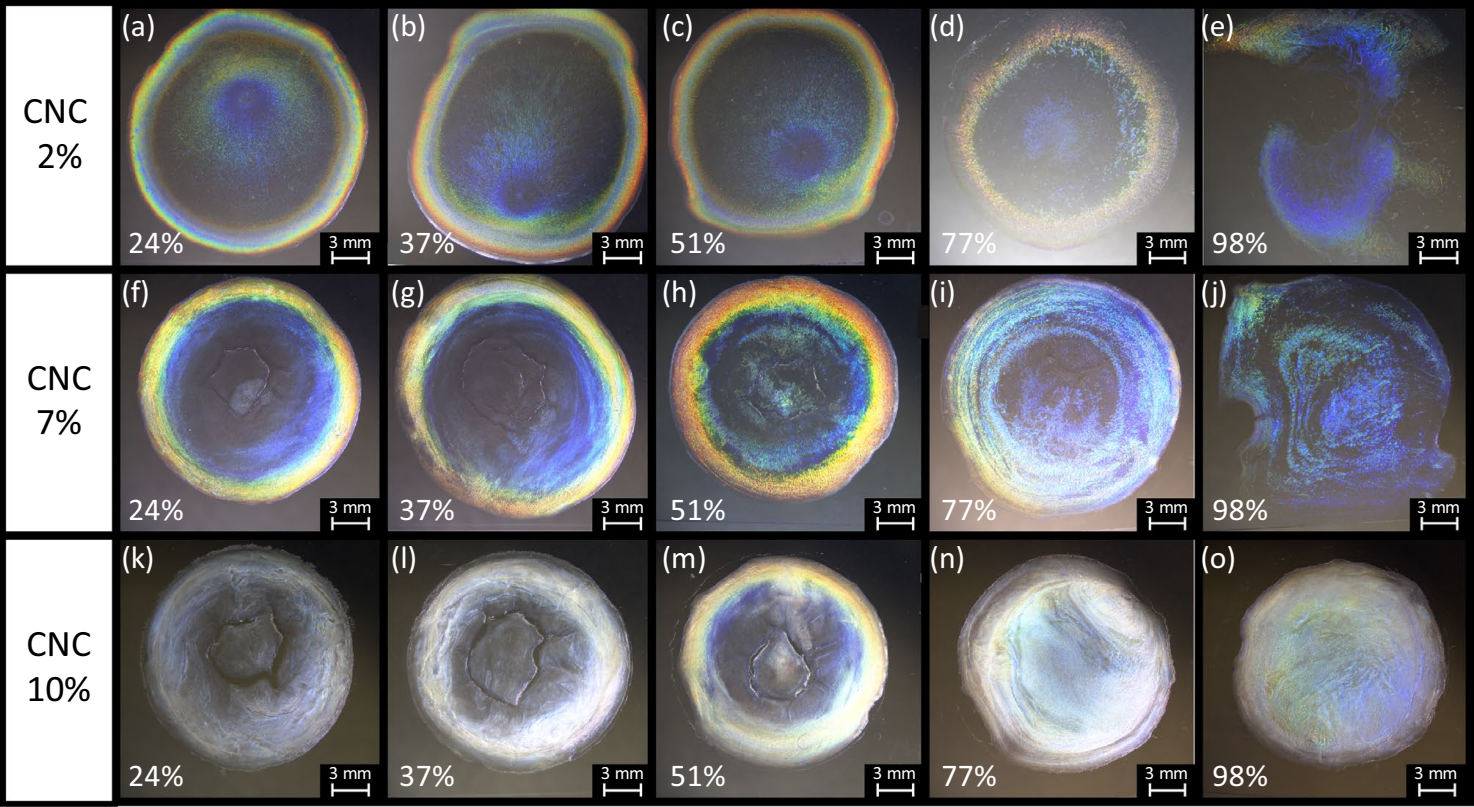
CNC films dried from droplets of (**a**)‒(**e**) 2%, (**f**)‒(**j**) 7%, and (**k**)‒(**o**) 10% CNC suspensions under increasing values of constant humidity (left to right). These images were acquired in reflection mode under white light without polarizers.

Humidity is another parameter that influences the rate of water evaporation from aqueous CNC suspensions and hence affects the quality of their films. The moisture content in air is defined as humidity, which is expressed as the ratio of the amount of water vapor in the air to that of the saturated water vapor at a certain temperature. Obviously, low humidity means that air can take up more water vapor, thus increasing the evaporation rate from the suspensions. On the other hand, high humidity means that air already contains a great deal of moisture; thus, water evaporates more slowly. Figure 5 compares films made from CNC suspension droplets dried under various constant humidities. As with the temperature-controlled films, the biphasic 7% CNC suspension exhibits the most iridescent texture among the films. As the controlled humidity increases, the coffee-ring stains extend further from the edge toward the inner part of the droplets. At the highest humidity, the blue color spreads widely and evenly at the center of the droplet.

In terms of iridescence, none of the films dried from droplets of the 2% and 10% CNC suspensions show sufficient color. However, weak coffee-ring stains in the films made from the 2% CNC suspension clearly tend to extend further toward the center of the droplet as the humidity increases, while appearing dark around the center of the droplet, which must be due to the isotropic nature of the 2% CNC suspension. Meanwhile, the droplets made from the 10% CNC suspensions appear bright whitish rather than as colorful coffee-ring stains, suggesting that mass transfer was inefficient during drying, presumably owing to the high viscosity of this concentrated suspension. In addition, this finding suggests that the helical pitch of the CNCs varies in the visible spectral region, and the CNC phase changes into a glassy state before reaching thermodynamic equilibrium.

As another way to control the rate of water evaporation, we also attempted to increase the ionic strength of CNC suspensions using NaCl^21^; however, adding NaCl drastically changed the suspensions, which immediately became opaque, indicating aggregation. The CNC suspensions must be in an electrostatically stabilized colloid state, wherein the delicate balance of the interaction potential among the colloids can be destroyed by changes in the ionic strength. In other words, the CNC suspensions were thermodynamically in a quasi-stable state. The energy barrier from the quasi-stable state to the globally stable state, namely, the aggregated state, can be lowered at higher ionic strengths.

**Figure 6:**
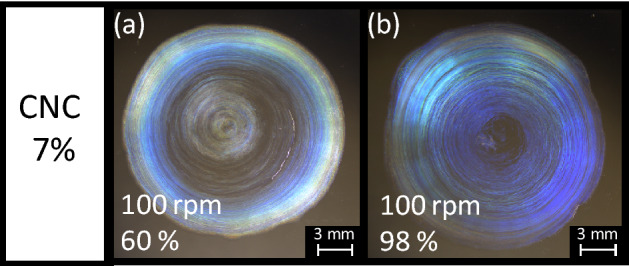
7% CNC droplet films dried under high humidity of (**a**) 60% and (**b**) 98% while being orbitally shaken anticlockwise. These images were acquired in reflection mode under white light without polarizers.

**Figure 7:**
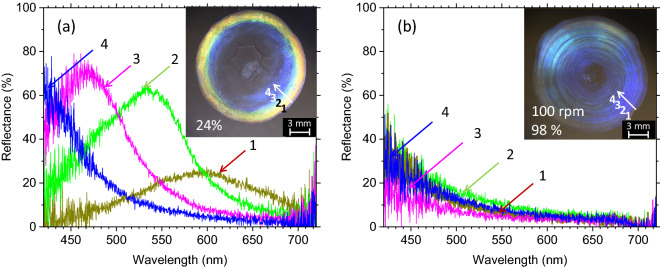
Reflection spectra of 7% CNC droplet films dried at (**a**) 24% humidity without orbital shaking and (**b**) 98% humidity while being orbitally shaken at an anticlockwise rotation speed of 100 rpm.

Further, to determine whether a more uniformly blue film can be attained, the humidity-controlled drying process was combined with orbital shaking, which is known to effectively form uniform CNC films^58^. Figure 6 shows two droplet films dried under two constant high humidity values while being orbitally shaken. The blue region more uniformly extends from the edge to the center of the droplet when combining high-humidity drying with orbital shaking, suggesting that these conditions indeed effectively enhance the uniformity of the blue region in the film. Figure 7 validates this uniformity by comparing the reflection spectra of two extreme cases: one is from the film made dried from the 7% CNC suspension without orbital shaking under 24% humidity, and the other formed from the same concentration during orbital shaking under 98% humidity. The latter indeed shows reflection peaks caused by selective reflection in the blue region at the positions from the edge to the center of the film, whereas reflections from the same positions in the former shift from the yellow to blue region.

As previously reported^58^, orbital flow may enable the distortion of tactoids, thereby forming microdomains of CNCs while breaking the symmetry of these tactoids such that their vertical helical axes are oriented. Although this process is plausible, another possible mechanism should also be considered, namely, that orbital flow breaks capillary flow. The latter is directly correlated with water evaporation. Drying under high humidity while being orbitally shaken uniformly enhances the effect of the slow evaporation over the entire area of the droplet. Indeed, under high-humidity drying combined with orbital shear, the blue region spreads over the entire film.

**Figure 8:**
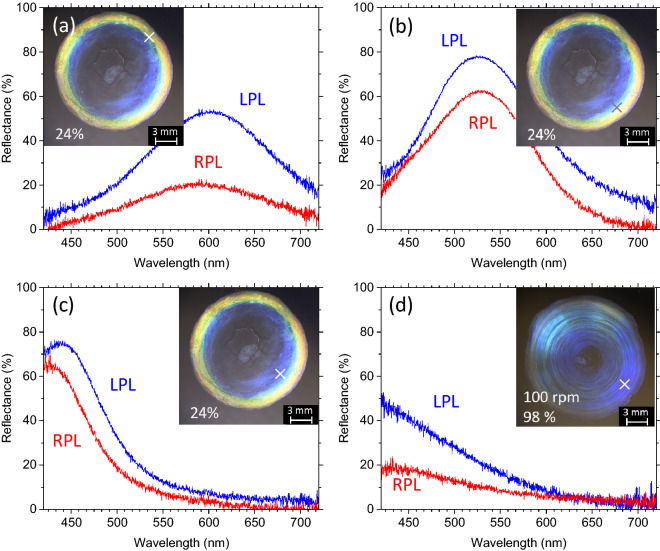
Reflection spectra recorded from points indicated by crosses in the insets under circularly polarized light showing various colors in 7% CNC droplet films dried at (**a**)‒(**c**) 24% humidity without orbital shaking and (**d**) 98% humidity while being orbitally shaken at 100 rpm. LPL and RPL are left- and right-handed circularly polarized light, respectively.

The various colors observed in the CNC droplet films from red to blue are indeed structural color originating from the periodic CNC structure, and these colors vary with the helical pitch in the CNC films. Figure 8 shows the reflection spectra of the same films presented in Fig. 7 but recorded using circularly polarized light as the input. Evidently, the left-handed helical pitch of the CNC reflects left-handed circularly polarized light (LPL) to a greater degree than it reflects right-handed polarized light (RPL). Unlike films of well-aligned cholesteric LCs, our CNC droplet films are obviously non-uniform in terms of thickness, helical pitch, and the orientations of their microdomains. Presumably, these intricate but non-uniform properties may depolarize the incoming light, and the LPL of the depolarized light would be preferentially reflected from the CNC films.

Our systematic observations above enable the development of a model to elucidate these results. One important finding is that the CNC helical pitch continually decreases as the CNC films form from the suspensions, suggesting that the helical pitch changes as water evaporates from the suspension droplets. In particular, temperature and humidity influence the rate at which water evaporates from aqueous suspensions, which in turn affects the CNC helical pitch, the dispersion of the CNC molecules, and the alignment of the helical axes in the films, as mentioned above. These considerations allow us to compare two types of LCs: thermotropic and lyotropic. In thermotropic LCs, the motions of rod-like and disk-like molecules in the fluid state can be frozen into a solid state depending on how rapidly the temperature decreases^70^. These materials can vitrify via the kinetic arrest of molecular motions in the fluid state, which dictates the degree of order in the resulting glassy state. The relationship between thermotropic LCs and temperature can be naturally observed in other common materials, including organic semiconductors, small molecules, and polymers; for example, good crystallinity and high molecular order both require a gradual decrease in temperature.

**Figure 9:**
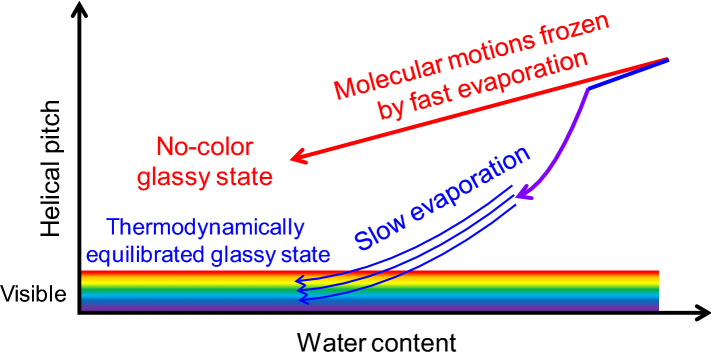
Qualitative model of the effects of fast and slow water evaporation on the helical pitch.

Analogously, the solvent (water in our case) plays a role akin to that of temperature in lyotropic LCs, including those formed in aqueous CNC suspensions. Figure 9 illustrates the qualitatively established model, wherein the water evaporation rate governs the degree of the helical pitch and molecular order. When water evaporates rapidly from the CNC suspension droplets, their helical pitch cannot be considered to change in a thermodynamically equilibrated manner^24,60^. In other words, CNCs can vitrify via the kinetic arrest^68,71–73^ of molecular motions in the fluid state. Considering that the formation of a uniformly colored film is based on the packing and alignment of molecules in the film, the molecular organization of CNCs, which are glass-forming LCs, can freeze during the drying process from the fluid state into a solid glass. By contrast, sufficiently slow water evaporation from the CNC suspension droplets can maintain thermodynamic equilibrium, and the CNC helical pitch shortens into the visible range while gradually following this equilibrium state, thereby exhibiting iridescence.

## Conclusions

Although attaining color uniformity in a CNC film remains a challenge, we have pursued this goal by investigating the conditions that yield iridescence and color uniformity in CNC films through the thermodynamically relaxed alignment and helical pitch of CNCs. The speed of water evaporation from a droplet is found to significantly affect the quality and uniformity of this iridescence. Slowly drying a droplet of a CNC-containing aqueous suspension enables the thermodynamic relaxation of the pitch into the blue region, and a relatively large-area domain can be attained via humidity-controlled drying. Rapid drying allows kinetic arrest to set in, and the thermodynamically non-equilibrium pitch varies from red at the edge to blue at the center of the droplet, presumably owing to capillary flow. A qualitative model of the drying process of aqueous CNC suspensions is conceived in which this kinetic arrest can prohibit the thermodynamic transition of the molecules to a stable pitch by freezing the molecular motions into a gel-like glassy state. This model can be understood by analogy to the kinetic arrest induced by the rate of temperature change in thermotropic LCs. With this model in mind, we can consistently interpret the coloration results in dried films formed at various temperatures and humidity levels. Considering the process of water evaporation, orbital shaking may play a role in breaking the capillary flow and spatially homogenizing the evaporation process from the surface of the aqueous CNC suspension droplets. To better understand our qualitative model, including the presumed role of orbital shaking, it would be important to quantify the rate of water evaporation and to spectroscopically or computationally probe the changes in the CNC structures during the water evaporation process. Thus, further efforts should be devoted to revealing the kinetic arrest induced by rapid water evaporation and achieving a uniformly colored CNC film while subduing this kinetic arrest.

## Data Availability

The datasets generated during and/or analyzed during the current study are available from the corresponding author on reasonable request.
